# Probing the Limits of Aptamer Affinity with a Microfluidic SELEX Platform

**DOI:** 10.1371/journal.pone.0027051

**Published:** 2011-11-14

**Authors:** Kareem M. Ahmad, Seung Soo Oh, Seon Kim, Forrest M. McClellen, Yi Xiao, H. Tom Soh

**Affiliations:** 1 Interdepartmental Program in Biomolecular Science and Engineering, University of California Santa Barbara, Santa Barbara, California, United States of America; 2 Materials Department, University of California Santa Barbara, Santa Barbara, California, United States of America; 3 Department of Electrical and Computer Engineering, University of California Santa Barbara, Santa Barbara, California, United States of America; 4 Chemistry and Biochemistry Department, University of California Santa Barbara, Santa Barbara, California, United States of America; 5 Department of Mechanical Engineering, University of California Santa Barbara, Santa Barbara, California, United States of America; University of Helsinki, Finland

## Abstract

Nucleic acid-based aptamers offer many potential advantages relative to antibodies and other protein-based affinity reagents, including facile chemical synthesis, reversible folding, improved thermal stability and lower cost. However, their selection requires significant time and resources and selections often fail to yield molecules with affinities sufficient for molecular diagnostics or therapeutics. Toward a selection technique that can efficiently and reproducibly generate high performance aptamers, we have developed a microfluidic selection process (M-SELEX) that can be used to obtain high affinity aptamers against diverse protein targets. Here, we isolated DNA aptamers against three protein targets with different isoelectric points (pI) using a common protocol. After only three rounds of selection, we discovered novel aptamer sequences that bind to platelet derived growth factor B (PDGF-BB; pI = 9.3) and thrombin (pI = 8.3) with respective dissociation constants (K_d_) of 0.028 nM and 0.33 nM, which are both superior to previously reported aptamers against these targets. In parallel, we discovered a new aptamer that binds to apolipoprotein E3 (ApoE; pI = 5.3) with a K_d_ of 3.1 nM. Furthermore, we observe that the net protein charge may exert influence on the affinity of the selected aptamers. To further explore this relationship, we performed selections against PDGF-BB under different pH conditions using the same selection protocol, and report an inverse correlation between protein charge and aptamer K_d_.

## Introduction

Aptamers [1], [2] are nucleic acid-based affinity reagents selected through an *in vitro* process that can bind to a range of molecular targets, including small molecules, proteins, and cells [3]. Aptamers offer a number of advantageous features, including low cost, high thermal stability and the capacity for chemical synthesis and modification, making them a promising alternative to antibodies and other protein-based reagents [4]. However, due to the iterative nature of the aptamer selection process (systematic evolution of ligands by exponential enrichment; SELEX), the generation of aptamers requires a significant investment of time, labor and resources [5], and selections often fail to yield molecules with affinities suitable for molecular diagnostics or therapeutics. Thus, a selection technique that can efficiently and reproducibly generate high performance aptamers is urgently needed.

Towards this end, our group recently reported a microfluidics-based approach for the rapid generation of DNA aptamers. Microfluidic SELEX (M-SELEX) exerts stringent selection pressures by employing minimal amounts of target molecules and continuous washing to isolate high affinity aptamers in fewer rounds of selection compared to conventional methods [6], [7], [8]. To extend this method, in this work, we report a universal M-SELEX process that can isolate high affinity aptamers for a range of protein targets with varying isoelectric points (pIs) within three selection rounds. Using a common selection protocol, we have discovered new aptamer sequences that bind to PDGF-BB [9] and thrombin [10], [11] with higher affinities than those previously published. We also report a new aptamer that binds apolipoprotein E3 [12], [13] with low nanomolar affinity. Based on the results obtained from these selections, we make the observation that target protein pI appears to be inversely related to the K_d_ of the selected aptamers. To further explore this relationship, we performed microfluidic selections with PDGF-BB under varying pH conditions to alter its charge state, and report differences in the affinities of the resulting aptamer pools.

## Results

### Microfluidic selection of aptamers

**Figure 1:**
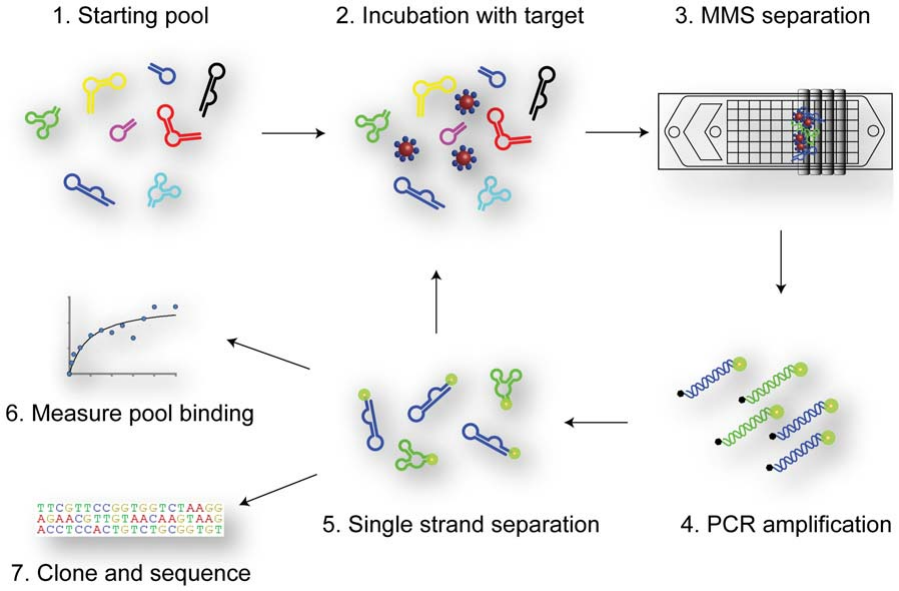
Schematic showing the major steps of M-SELEX. The starting pool (1) is incubated with magnetic beads coated with the target protein (2). After incubation, the sample is loaded onto the micro-magnetic separation (MMS) device, which traps the beads for high-stringency washing (3). The eluted beads are then PCR-amplified to generate double-stranded DNA (4). The fluorescently-labeled forward strand is recovered during a single-strand separation step (5), and can then be used for the next selection round, binding measurements (6) or cloning and sequencing (7).

We performed DNA aptamer selections for three separate protein targets under the same selection conditions. For every round of selection, we used the micro-magnetic separation (MMS) device [6] to efficiently separate non-specifically-bound DNA from target-bound aptamers. For each target, we performed three rounds of selection with increasing stringency (Fig. 1). This stringency was controlled in two ways. First, we used a low concentration of target protein during incubation and decreased this concentration after the first round of selection. Second, we used a high flow rate during MMS washing and increased the wash time to remove weakly- and non-specifically-bound DNA (see Methods).

**Figure 2:**
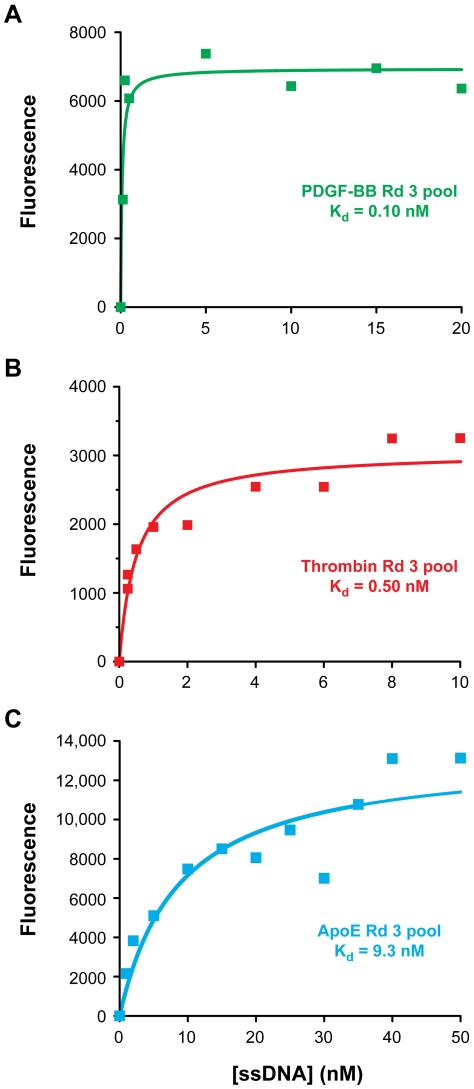
Affinities of round 3 pools for three target proteins. Selections performed against three different proteins yielded enriched aptamer pools whose binding affinities were dependent on pI as determined by a bead-based fluorescence assay. (A) PDGF-BB round 3 pool K_d_ = 0.10 nM. (B) Thrombin round 3 pool K_d_ = 0.50 nM. (C) ApoE round 3 pool K_d_ = 9.3 nM.

After three rounds of selection, the affinities of the enriched aptamer pools were measured by a bead-based fluorescence assay [6]. The selected pool for PDGF-BB (pI = 9.3) showed the highest affinity (K_d,bulk_ = 0.10 nM) (Fig. 2A); the thrombin pool (pI = 8.3) showed moderate affinity (K_d,bulk_ = 0.50 nM) (Fig. 2B); and the ApoE pool (pI = 5.3) displayed the lowest affinity (K_d,bulk_ = 9.3 nM) (Fig. 2C). These selection results reveal a clear correlation between the bulk binding affinity of the enriched pools and target protein isoelectric point (Fig. 2).

### Identification of individual binding sequences

To obtain individual aptamer sequences for each target protein, we cloned the round 3 pools into competent bacterial cells, and randomly picked and sequenced twenty clones from each pool (Table S1) using a method described previously [6]. We found that the sequences for PDGF-BB and ApoE were all unique, indicating that the round 3 pools were not fully converged. On the other hand, with the exception of three orphan sequences, all of the thrombin sequences belonged to one of two nearly identical consensus groups, indicating that the pool had almost fully converged. This differential rate of sequence convergence has also been previously described. For example, the Bock thrombin aptamer was obtained after only five rounds of selection [10], while the Green PDGF aptamer required 12 rounds of selection [9]. Despite differences in selection methods, this apparent difference in convergence rates for individual targets suggests that this phenomenon may arise from characteristics of the target protein itself.

**Figure 3:**
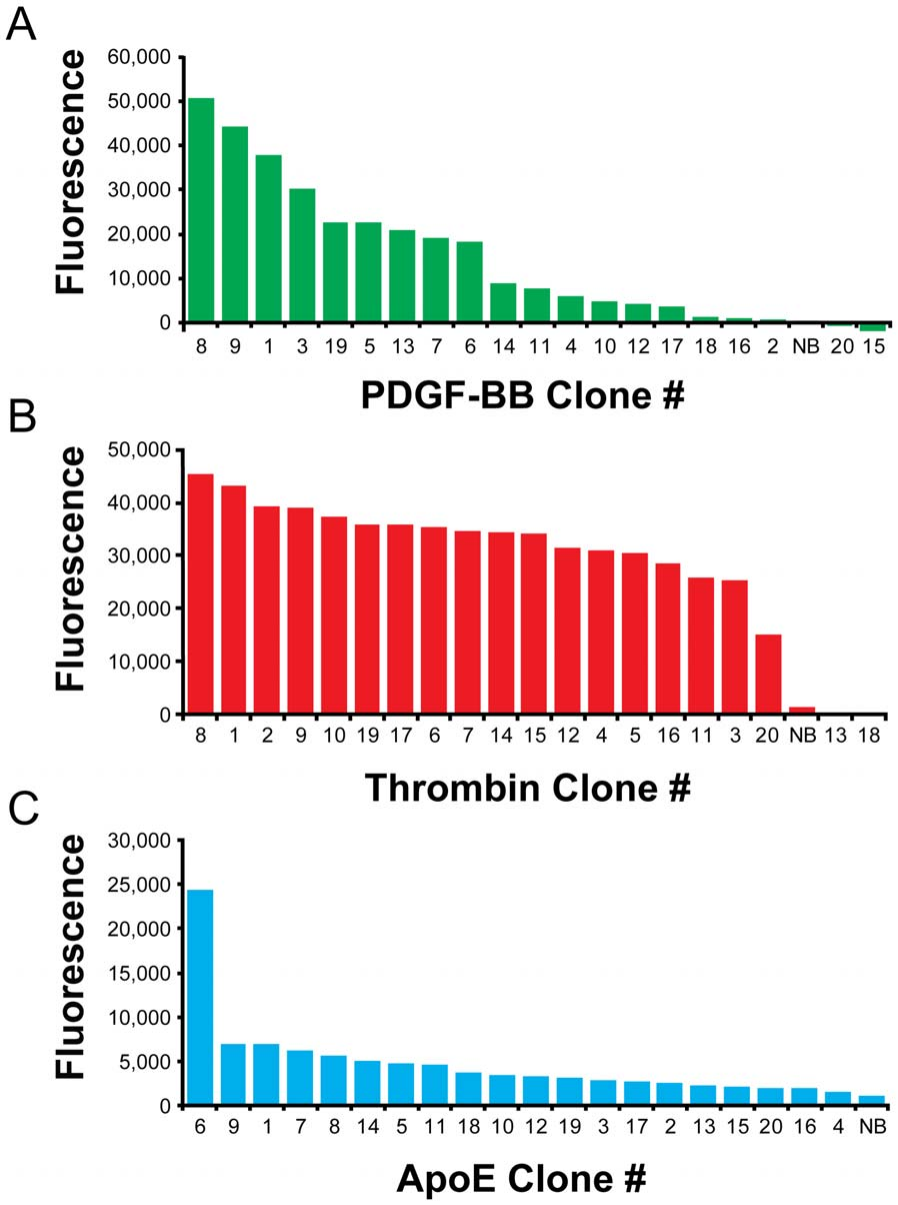
Screening of clones for target binding. We quantified the relative binding affinities of 20 clones from each selection using a bead-based fluorescence binding assay at a single ssDNA concentration. NB indicates a non-binding control sequence included in each measurement. We chose the top binding sequences for further analysis and determination of equilibrium dissociation binding constant (K_d_). (A) PDGF-BB with [ssDNA] = 0.50 nM, NB = Thrombin Clone #1. (B) Thrombin with [ssDNA] = 0.50 nM, NB = PDGF Clone #1. (C) ApoE with [ssDNA] = 35 nM, NB = Thrombin Clone #1.

To rapidly identify the sequences with the highest affinities, we measured the relative binding of each clone at a fixed concentration with a bead-based fluorescence binding assay, and rank-ordered the clones based on signal amplitude (Fig. 3). We generated fluorescently-labeled sequences by PCR with an Alexa Fluor 488-labeled forward primer, followed by lambda exonuclease digestion. Based on the bulk binding affinities of the three pools, we used a concentration of 0.5 nM to measure the PDGF-BB and thrombin sequences, and 35 nM for the ApoE sequences. The PDGF-BB clones showed a wide range of relative binding affinities, reflecting the diversity of these sequences (Fig. 3A). The round 3 thrombin pool was highly converged and, as expected, all of the thrombin clones showed strong affinity except for two of the orphan sequences (Fig. 3B). Interestingly, a single ApoE clone showed significantly greater affinity compared to the other clones at the concentration tested (Fig. 3C).

### Characterization of selected sequences

**Figure 4:**
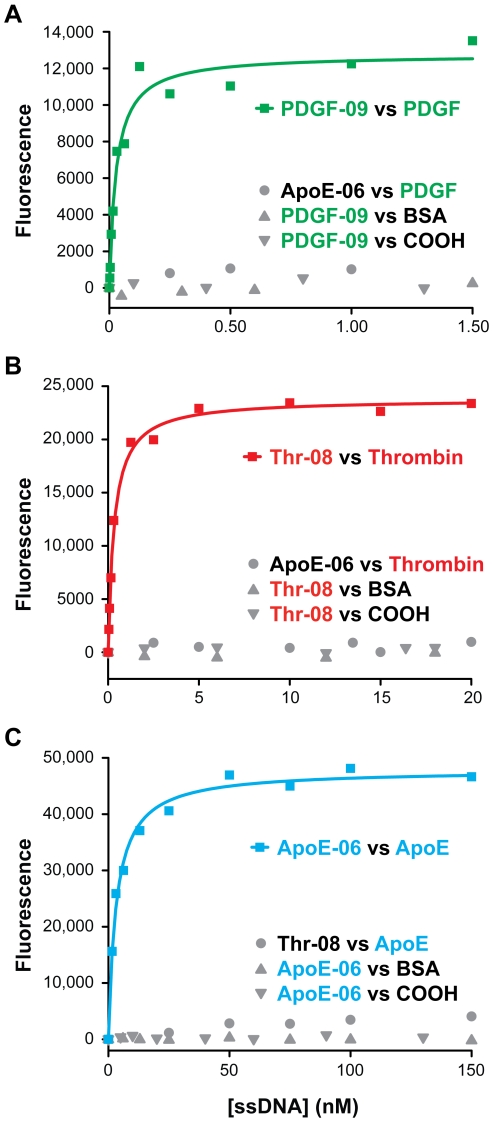
Affinity measurements of individual aptamer sequences for each protein target. (A) PDGF-09 binds to PDGF-BB with K_d_ = 0.028 nM, with negligible binding to bovine serum albumin-coated (BSA) or uncoated beads (COOH). As a negative control, we measured the binding of ApoE-06 to PDGF-BB. (B) Thr-08 binds to thrombin with K_d_ = 0.33 nM and shows negligible binding to BSA-coated or uncoated beads. Binding of ApoE-06 to thrombin is minimal. (C) ApoE-06 binds to ApoE with K_d_ = 3.1 nM and shows negligible binding to BSA-coated or uncoated beads. Thr-08 has minimal affinity for ApoE.

We measured the K_d_ values of the sequences that exhibited the highest relative binding: the three top sequences for PDGF-BB, one sequence from each of the two consensus groups for thrombin and the top binding ApoE sequence. We obtained binding isotherms by the same bead-based fluorescence binding assay described above, and fit the results by non-linear regression (Table 1). These sequences all exhibited high affinities for their targets and negligible binding to non-target proteins and uncoated carboxylic acid beads (COOH) (Fig. 4). For example, our PDGF-BB aptamer (PDGF-09) binds with a K_d_ of 0.028 nM (Fig. 4A) which is significantly better than the previously reported aptamer, which was obtained after 12 rounds of selection and reported to have a K_d_ of 0.093 nM based on a filter-binding assay [9]. To directly compare, we measured the affinity of this previously-reported aptamer (Table S2) using our bead-based fluorescence assay and obtained a K_d_ of 0.12 nM (Fig. S1).

**Table 1:** The sequences of the top-binding aptamers and their affinities.

Clone ID	Selected region (5′ to 3′)	K_d_ (nM)
PDGF-01	CACGCGTACAAGTTGGGTGGAAGCATAGGCAATGAGCTCTCATTGGGTTACCTTTAAGGT	0.10
PDGF-08	GTTGCATATTGAGCATCGTGTAATGATCCTGCAAAGGCATTATGCATCGGGTCTTCCT	0.30
PDGF-09	GAATCATGATCCCCCATGACCTGGGGGCGTTACTGGCGAGCATCATGAGAGATCGTGCGA	0.028
Thr-02	TTGAAGTAATTTTATAGGTCTTTTATTGGGTAGGGTGGTTTTAATTGGTGTGACAAATTG	0.65
Thr-08	CAGCGCTAGGGCTTTTAGCGTAATGGGTAGGGTGGTGCGGTGCAGATATCGGAATTGGTG	0.33
ApoE-06	ACTAGCTACGGGGTGGGTGGGCGGTGTCAGTTTGTTTATTGGTGCTATACATCCTCTATA	3.1

Only the variable sequence (60-mer) is shown in the table, but affinity and specificity measurements were performed with full-length (100-mer) molecules containing the flanking PCR primer-binding sites.

The highest-affinity thrombin aptamer sequence (Thr-08) obtained in this selection exhibits a K_d_ of 0.33 nM (Fig. 4B). The affinity of this aptamer is better than those previously reported by Bock [10] and Tasset [11] under the same measurement conditions (Table S2). Using our assay, the Bock and Tasset aptamers showed K_d_s of 1.2 nM and 11.9 nM, respectively (Fig. S2). The ApoE aptamer binds with a K_d_ of 3.1 nM (Fig. 4C), and this sequence represents the first reported DNA aptamer for this protein. To confirm the bead-based binding measurements of the three aptamers, we performed binding measurements using nitrocellulose filters with ^32^P-labeled aptamers (Fig. S3). The results of this solution-based method show reasonable agreement with those of the bead-based assay.

### Charge state of the target protein and aptamer affinity

Interestingly, when we examined the affinity of the selected aptamers as a function of the isoelectric point (pI) of the target protein, we observed a trend wherein aptamer affinity increased with target pI. For example, the aptamer obtained in the selection against ApoE, which has a pI of 5.3 and net negative charge, displayed the lowest target affinity. The aptamer affinity was somewhat higher for thrombin, a protein with a near-neutral pI and small net positive charge, while aptamers against PDGF-BB, which has the highest pI (9.3) and most positive net charge of the three targets, exhibited the highest affinity. pIs were determined computationally (see Methods) and then experimentally verified using isoelectric focusing (Fig. S4).

**Figure 5:**
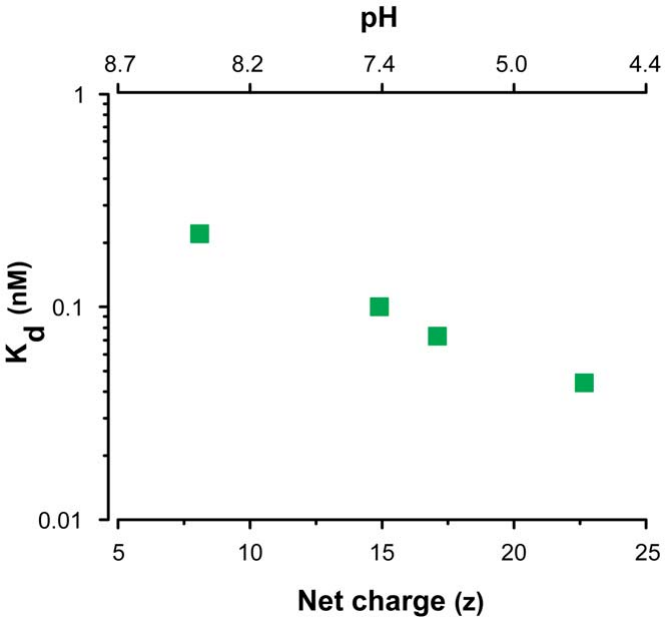
Affinity of selected aptamer pools depends on protein charge. The binding affinities (K_d_) of selected round 3 aptamer pools are shown as a function of the pH of the selection buffer (pH = 4.6, 6.4, 7.4 and 8.4). Selection at pH 4.6 (z = +22.7) generated an aptamer pool with the highest affinity (K_d_ = 0.044 nM) whereas selection at pH 8.4 (z = +8.1) yielded the pool with the lowest affinity (K_d_ = 0.22 nM). Calculations of net charge as a function of pH are described in Methods.

Our capacity for rapid selection and affinity measurements enabled us to further explore the relationship between the target protein charge state and the affinity of the selected aptamers. We performed three additional selections with PDGF-BB using selection buffers spanning a range of pHs (4.6, 6.4, and 8.4) to alter the net charge on the protein. The results show a clear link between the net charge and the affinity of the resulting round 3 aptamer pool (Fig. 5). Selection at the higher pH of 8.4 (z = +8.1), yielded an aptamer pool with lower affinity (K_d_ = 0.22 nM) relative to the selection performed at the physiological pH of 7.4 (z = +14.9; K_d_ = 0.10 nM). On the other hand, the selection performed at the lowest pH, 4.6 (z = +22.7), yielded the aptamer pool with the highest affinity (K_d_ = 0.044 nM). These results provide evidence that a protein target's net charge may influence the affinity of the selected aptamers.

## Discussion

In this work, we have demonstrated that M-SELEX provides a highly efficient and broadly applicable platform for the selection of high-affinity aptamers against a variety of protein targets. Using this method, we performed selections against three different proteins with pIs ranging from 9.3 to 5.3. Within three rounds of selection, we obtained a new aptamer sequence that binds to PDGF-BB with a K_d_ of 0.028 nM, and to thrombin with a K_d_ of 0.33 nM. These affinities are significantly higher than those previously reported in the literature [9], [10], [11], which also required considerably more rounds of selection to generate. We also report a new aptamer targeting ApoE with a K_d_ of 3.1 nM.

The results obtained with these three targets suggest that the target protein charge state may exert some degree of influence on the overall affinity of the selected aptamer pool. To follow up on this finding, we performed aptamer selections for PDGF-BB at different buffer pHs using the same selection protocol which revealed an apparent inverse correlation between protein pI and aptamer K_d_ and therefore provided additional support for this initial observation.

**Figure 6:**
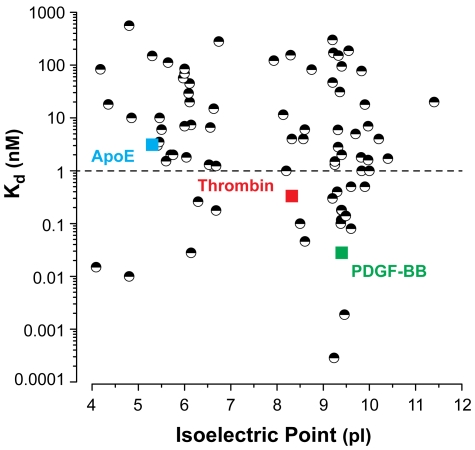
Aptamer affinity versus isoelectric point. A plot of equilibrium dissociation constant (K_d_) vs. isoelectric point (pI) for 75 published aptamers and the three newly discovered sequences from this work. We observe an apparent inverse relationship between protein pI and aptamer K_d_. The proteins were identified in the UniProt database and isoelectric points were determined from the Compute pI/Mw tool at ExPASy.

To explore whether this relationship between a protein's charge state and aptamer affinity potentially represents part of a broader pattern, we plotted the K_d_s of 75 published aptamers (Table S3) as a function of the pI of their target protein (Fig. 6). pI reflects the average net charge of a protein and does not describe the spatial charge distribution, but nevertheless offers a useful metric to make a general observation of a trend. In this scatter plot, the relationship between aptamer affinity and the protein's isoelectric point is not immediately obvious, presumably due to the differences in selection conditions and variability in affinity measurement methods.

However, when we examined only the subset of aptamers with K_d_<1 nM, we observed a clear trend. For targets with pIs ranging from 4.0 to 6.0, only 11% (2/19) of the resulting aptamers exhibited subnanomolar affinity, but this percentage increased to 23% (3/13) for aptamers isolated against targets with pIs ranging from 6.0 to 8.0 and to 35% (15/43) for aptamers against proteins with a pI greater than 8.0. Based on this analysis, we observe additional evidence for a relationship between the overall net charge of a target protein and the affinity of aptamers selected against that target.

Electrostatic surface potential maps of the three protein targets selected here offer a potential explanation for this trend (Figure S5); the protein with the highest proportion of positively-charged surface area (PDGF-BB) yielded the highest affinity aptamer, whereas the protein with the lowest proportion (ApoE) yielded the lowest affinity aptamer. However, other physicochemical factors such as size, structural complexity and intrinsic stability likely play important roles in governing protein-aptamer interactions. Further progress in the development of rapid selection techniques such as the microfluidic approach described here should make it possible to explore the relative impact of these target protein characteristics in greater detail. In parallel, recent work exploring the incorporation of non-natural bases into aptamers [14], [15] suggests additional means by which one might use selection strategies such as this to obtain molecules with yet higher affinity for an even broader range of protein targets.

## Materials and Methods

### DNA preparation

The single-stranded DNA (ssDNA) library, primers and selected aptamer sequences were purchased from Integrated DNA Technologies. The library was synthesized with hand mixing and PAGE-purified. Each element of the 100-base random library featured 60 randomized nucleotides flanked by two 20-base PCR primer sites (5′-AGCAGCACAGAGGTCAGATG-[60N]-CCTATGCGTGCTACCGTGAA-3′). Unlabeled and 5′-modified PCR primers were obtained from Integrated DNA Technologies with HPLC purification. Fluorescein amidite (FAM)-labeled versions of previously published aptamers (Table S2) were obtained from Biosearch Technologies with HPLC purification.

### Target preparation and verification

All proteins except thrombin (purchased from Haematologic Technologies) were purchased from R&D Systems (carrier-free), reconstituted in the appropriate selection buffer, and stored at temperatures specified by the manufacturers. Protein immobilization on M-270 carboxylic acid beads (Invitrogen) was performed as described previously [6]. Surface coverage of the immobilized protein was measured by the NanoOrange Protein Quantitation kit (Invitrogen). The included bovine serum albumin (BSA) standard was used to construct a calibration curve for each of the three target proteins. Samples of Tris-blocked beads were assayed in parallel, and those fluorescence values were subtracted from the values obtained from the protein-coated beads. This value was then converted to a protein concentration based on the BSA calibration curve. To verify successful coupling, we measured the protein concentration of the solution before and after coupling.

### Device fabrication

The micro-magnetic separation (MMS) device was previously developed by our group [6], [16]. Briefly, the device was fabricated on borosilicate glass substrates with 25-µm-thick double-coated tape (3 M). The micropattern was defined on the bottom glass substrate with 20-nm-thick titanium and 200-nm-thick nickel films using standard photolithography methods. Pitch distances of 200, 100, and 50 µm were used in the nickel grid pattern to provide increasing grid density. Fluidic vias were drilled through the glass substrates using a computer-controlled CNC mill and diamond bit (Abrasive Technology). After cleaning with acetone, the Ti/Ni layer was passivated with a 100-nm-thick layer of SiO_2_ by plasma-enhanced chemical vapor deposition (Plasma-Therm). Microfluidic channels were cut out of the 25-µm-thick double-sided tape using a plotting cutter (Graphtec). The patterned tape was overlaid onto the top glass substrate manually. The channel and micropattern were then manually aligned, and the device was cured in an oven at 70°C overnight with light clamping pressure. A brass eyelet was used as the buffer inlet, and Tygon tubing (Saint-Gobain) was used for the sample inlet and outlet fluidic connections. All connections were glued in place using five-minute epoxy (Devcon). The external magnets consisted of eight stacked neodymium magnets (K&J Magnetics), with another magnet of the same type sandwiching the device to secure it during the separation and washing steps.

### Microfluidic SELEX

Aptamer selection for platelet-derived growth factor BB (PDGF-BB) and apolipoprotein E3 (ApoE) was performed in phosphate-buffered saline (PBS, pH 7.4) supplemented with 1 mM MgCl_2_ and 0.025% Tween 20. For the thrombin selection, the buffer consisted of 50 mM Tris, 100 mM NaCl, 1 mM MgCl_2_, 5 mM KCl, 1 mM CaCl_2_ and 0.025% Tween 20 at pH 7.5.

Before selection, target-coated beads were washed with selection buffer three times. For the first round of SELEX, 1 nmol (equivalent to ∼6×10^14^ molecules) of library was heated to 95°C for 10 min, snap-cooled on ice, then brought to room temperature and incubated with washed beads for 1 hour with gentle rotation. Approximately 10^6^ beads were used for the first round, and half that amount for the second and third rounds. In rounds 2 and 3, target-coated beads were blocked for 1 hour with 0.1 mM yeast tRNA (Invitrogen), then incubated for 1 hour with 100 pmole of snap-cooled ssDNA generated from the previous round.

Following incubation, the sample was loaded into the MMS device at 7 mL/hr through the top inlet with buffer flowing through the bottom inlet at 3 mL/hr. The beads were then washed at a combined flow rate of 50 mL/hr with selection buffer for 6 minutes in the first round and 20 minutes in rounds 2 and 3, after which the aptamer-bound beads were eluted from the MMS device with 800 µL of selection buffer. The configuration of the MMS device and pumps is shown in Figure S6. 10 µL of eluate were amplified by pilot PCR to determine the optimal number of rounds required for amplification without accumulating amplification byproduct [6]. The eluted DNA was then PCR amplified using a biotinylated reverse primer. This double-stranded DNA (dsDNA) was purified with Qiagen minElute columns and the concentration was measured by UV spectroscopy. Finally, we generated ssDNA using the streptavidin-biotin method described previously [6], [7], measured concentration by UV spectroscopy and verified purity on a 4.5% low-melt agarose gel pre-stained with GelStar (Lonza). For DNA pools that were to be assayed for affinity, we used 5′-Alexa Fluor 488-labeled forward primers for PCR amplification. The selections against PDGF-BB at varying pHs were performed as above, and buffer components were identical except for the pH 4.6 selection, in which the buffering agent was acetate.

### Cloning and sequencing of selected aptamers

A small amount of DNA (250 pg) was amplified with unlabeled primers, purified and cloned into chemically competent *E. coli* using the TOPO TA Cloning Kit (Invitrogen). The transformed bacteria were plated out and grown overnight at 37°C. Bacterial colonies were sent for sequencing at GeneWiz. Individual clones were amplified using a phosphorylated reverse primer and Alexa Fluor 488-modified forward primer, then purified as above and subjected to lambda exonuclease (New England Biosciences) digestion to remove the phosphorylated strand and produce ssDNA [17]. The resulting ssDNA was purified by phenol/chloroform extraction and ethanol precipitated. ssDNA was resuspended in Millipore water and its concentration measured by UV spectroscopy. The purity was confirmed on a 4.5% low-melt agarose gel stained with GelStar.

### Characterization of aptamer binding affinity by fluorescence bead assay

To measure the binding affinities of the selected pools and sequences, we incubated a range of concentrations of Alexa Fluor 488-labeled ssDNA with target-coated beads for 1 hour at room temperature with gentle rotation. Each sample was then washed four times using a Magnetic Particle Concentrator (Invitrogen), and the remaining bound DNA was eluted by incubation in selection buffer at 95°C for 10 minutes. The amount of fluorescence in the supernatant was measured with a microplate reader (Tecan) and the background subtracted fluorescence values were fit to a saturation binding curve using nonlinear regression (assuming 1∶1 binding) with Origin software (OriginLab). The top binding clones in each selection were synthesized by Integrated DNA Technologies with PAGE purification and prepared by PCR for binding measurements with Alexa Fluor 488-labeled forward primer and phosphorylated reverse primer. Exonuclease digestion, subsequent purification, and DNA purity confirmation steps were performed as described above.

### Characterization of aptamer binding affinity by filter binding assay

ssDNA to be assayed was end-labeled with [γ-^32^P]ATP (Perkin Elmer) using T4 polynucleotide kinase (New England Biolabs) and then purified with a G-25 spin column (GE Health Sciences). We estimated the protein concentrations by absorbance at 280 nm and calculated the extinction coefficients using ProtParam (web.expasy.org/protparam/). We used mixed ester filters (Millipore) for binding experiments with the thrombin and PDGF-BB sequences. End-labeled DNA was snap cooled then incubated in selection buffer with a range of protein concentrations for one hour at room temperature with gentle rotation. The samples were then pushed through pre-wetted filters mounted on holders (Millipore). The filters were washed with 5 mL of buffer then dried with 1 mL of air. We measured the radioactivity of the initial sample, flow through, washes, and filter with a liquid scintillation counter (Beckman Coulter). For the apolipoprotein E aptamer, we used polyvinylidene fluoride (PVDF) membranes (GE Healthcare) and performed the incubation in two steps. First, protein solution was passed through the pre-wetted membrane and then the end-labeled DNA was incubated with the membrane for one hour. Background subtracted counts per minute (CPM) values were fitted to a saturation binding curve using nonlinear regression (assuming 1∶1 binding) with Origin software (OriginLab).

### Calculating and measuring protein isoelectric point

For the three proteins selected here (PDGF, thrombin and ApoE), we calculated the pI and charge using pK_a_ values from the European Molecular Biology Open Software Suite (EMBOSS) based on amino acid sequences obtained from UniProt (www.uniprot.org). pIs of previously published aptamer protein targets were determined from the Compute pI/Mw tool at ExPASy (web.expasy.org/compute_pi/) using sequence information from UniProt.

Protein pIs were measured by isoelectric focusing (IEF). Pre-cast, 24 cm IEF gels were purchased from GE Healthcare and rehydrated with IPG buffer (GE Healthcare) and 1 µg of the protein being measured according to the manufacturer's instructions. The gels were run for 14.5 hours at 500 V and for 8.5 hours at 3500 V on a Multiphor II electrophoresis system (GE Healthcare). Afterwards, gels were stained with Sypro Ruby (Invitrogen) and imaged using UV illumination. Band distances were determined using ImageJ and corresponding pIs were calculated from a piece-wise linear fit of the manufacturer's calibration curves.

### Generation of electrostatic surfaces

Protein surface potentials were generated using PDB2PQR (kryptonite.nbcr.net/pdb2pqr/) and the Adaptive Poisson-Boltzmann Solver (APBS) [18], and plotted with VMD [19]. Protonation states were determined using the computed net charges and ionic conditions of the selection buffer and the PARSE generated surface potentials were mapped to the van der Waals surfaces. Thrombin structure 3F68 is shown for the protein only without the ligand. 1PDG (PDGF-BB) is shown in dimer form. 2L7B was used for ApoE.

## Supporting Information

Figure S1 **Affinity of a previously published aptamer to PDGF-BB.** Binding affinity of the previously published Green aptamer [9] as measured using our bead-based fluorescence assay. We obtained a K_d_ of 0.12 nM, which is similar to the original published value (0.093 nM, based on a filter-binding assay).
(TIF)

Figure S2 **Affinity of previously published aptamers to thrombin.** Bead-based fluorescence binding data of previously published Bock [10] and Tasset [11] thrombin aptamers showing measured K_d_s of 1.2 nM (**A**) and 11.9 nM (**B**), respectively.
(TIF)

Figure S3 **Affinity of selected radiolabeled aptamers based on filter binding.** We determined the binding affinity of the three selected aptamers by a filter binding assay. We measured K_d_s for PDGF-BB-, thrombin- and ApoE-binding aptamers of 0.106 nM (A), 0.936 nM (B) and 9.25 nM (C), respectively.
(TIF)

Figure S4 **Measuring the isoelectric points (pIs) of PDGF-BB, thrombin and ApoE.** We used isoelectric focusing (IEF) to measure target protein pI directly for (A) PDGF-BB, (B) thrombin and (C) ApoE under denaturing conditions for comparison against calculated pIs. pIs were determined based on band distance from the anodic end of the gel after focusing. For thrombin, we observed two main bands in the region from 7 to 7.5, in agreement with previous results [20].
(TIF)

Figure S5 **Electrostatic potential surfaces of PDGF-BB, thrombin and ApoE.** The electrostatic potentials are mapped to the van der Waals surfaces of these three proteins. The bottom set of views show the proteins rotated 180° about the vertical axis. These three maps suggest a structural basis for the relationship between charge and binding affinity. Reflecting its high net positive charge (z = +15), a substantial portion of the PDGF-BB homodimer surface has a highly positive surface potential. These regions present sizable areas for possible interactions with the highly negatively charged DNA aptamers, and were identified as the actual binding sites for a previously-isolated PDGF-AB aptamer through cross-linking experiments [9]. Thrombin (z = +2.6) contains two somewhat smaller distinct regions of positive surface potential, and these were also identified as the binding sites of two previously-published thrombin aptamers [10], [11]. The surface potential of ApoE (z = −4.3) is more heterogeneous, reducing the area available for aptamer binding. These data support the view that aptamers often preferentially bind to positively charged surface patches, as has been observed in crystal structures of aptamers bound to proteins [21], [22], [23], [24], [25], [26].
(TIF)

Figure S6 **Schematic of micro-magnetic separation (MMS) device and pump configuration.** The organization of the fluidic connections between the pumps and MMS chip are shown. Syringe pumps (not shown) were used to infuse buffer solution.
(TIF)

Table S1 **Selected sequences.** 60-mer random regions cloned from each selected round 3 pool.
(DOCX)

Table S2 **Sequences of previously published aptamers.** We synthesized these aptamer sequences with a 5′-FAM fluorophore and assayed their binding affinity using the bead-based method.
(DOCX)

Table S3 **A list of 75 previously published aptamers.** This list was compiled from PubMed searches and includes both RNA and DNA aptamers against protein targets. Where not provided in the reference, isoelectric points were determined by UniProt (www.uniprot.org) and ExPASy (www.expasy.org).
(DOCX)
