# Supplementary figures and images for: Probing the Limits of Aptamer Affinity with a Microfluidic SELEX Platform
Source: PLoS One. 2011 Nov 14;6(11):e27051. doi: 10.1371/journal.pone.0027051 (PMC3215713; doi:10.1371/journal.pone.0027051)

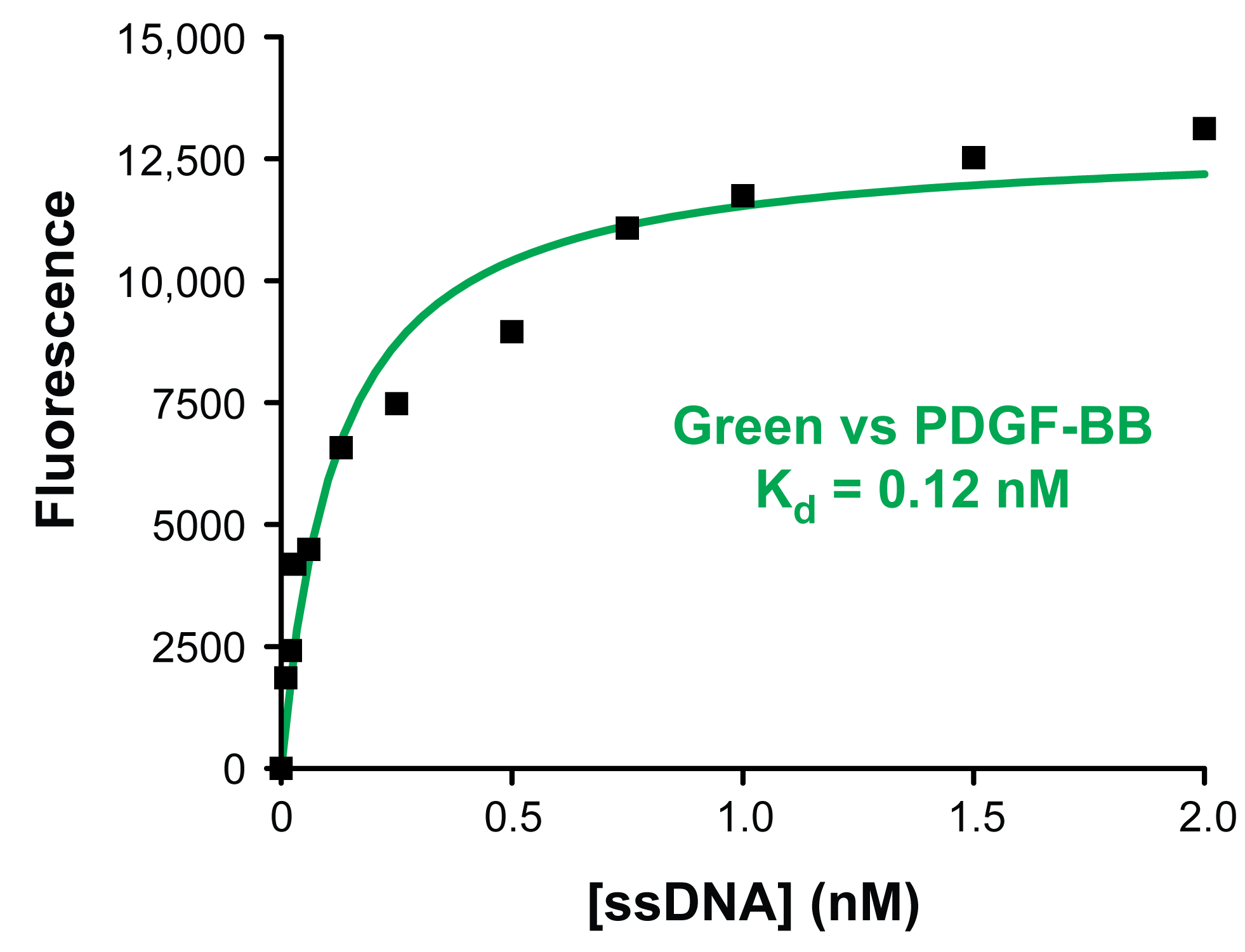

Supplement: Figure S1 — Affinity of a previously published aptamer to PDGF-BB. Binding affinity of the previously published Green aptamer [9] as measured using our bead-based fluorescence assay. We obtained a Kd of 0.12 nM, which is similar to the original published value (0.093 nM, based on a filter-binding assay). (TIF) [file pone.0027051.s001.tif]

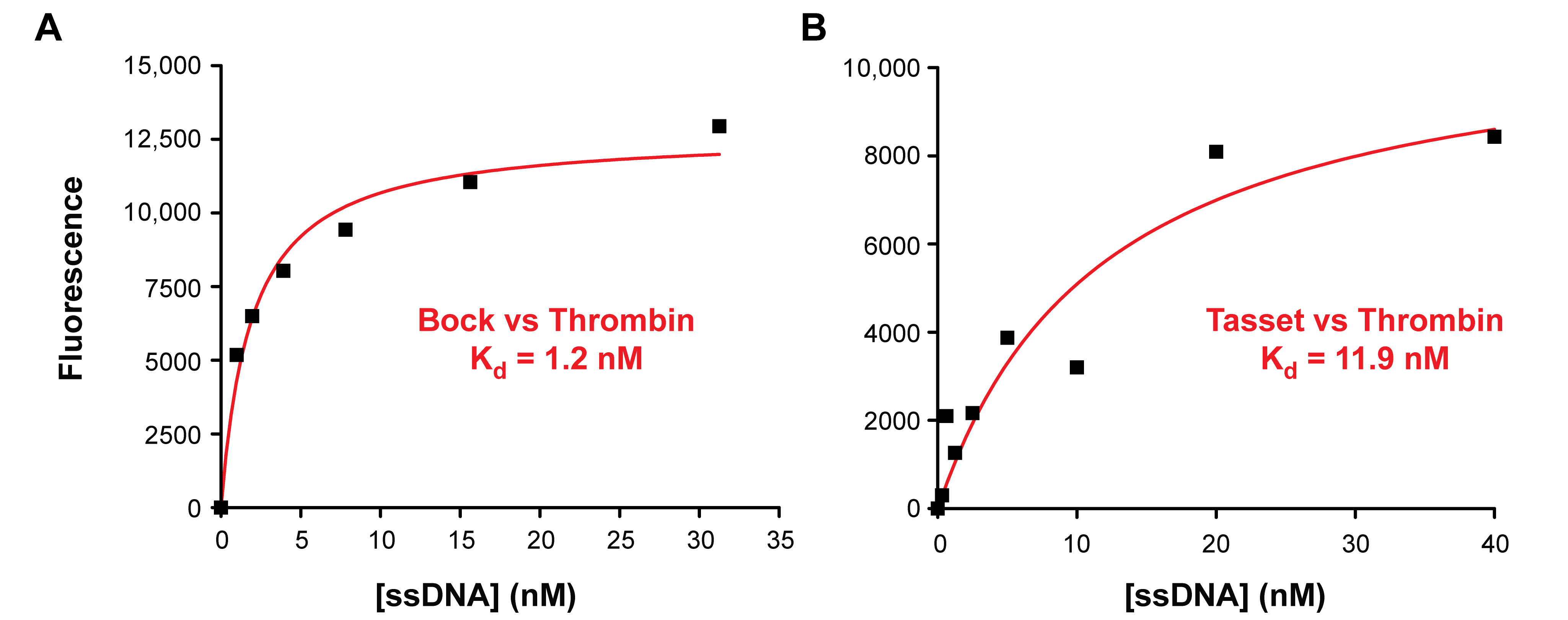

Supplement: Figure S2 — Affinity of previously published aptamers to thrombin. Bead-based fluorescence binding data of previously published Bock [10] and Tasset [11] thrombin aptamers showing measured Kds of 1.2 nM (A) and 11.9 nM (B), respectively. (TIF) [file pone.0027051.s002.tif]

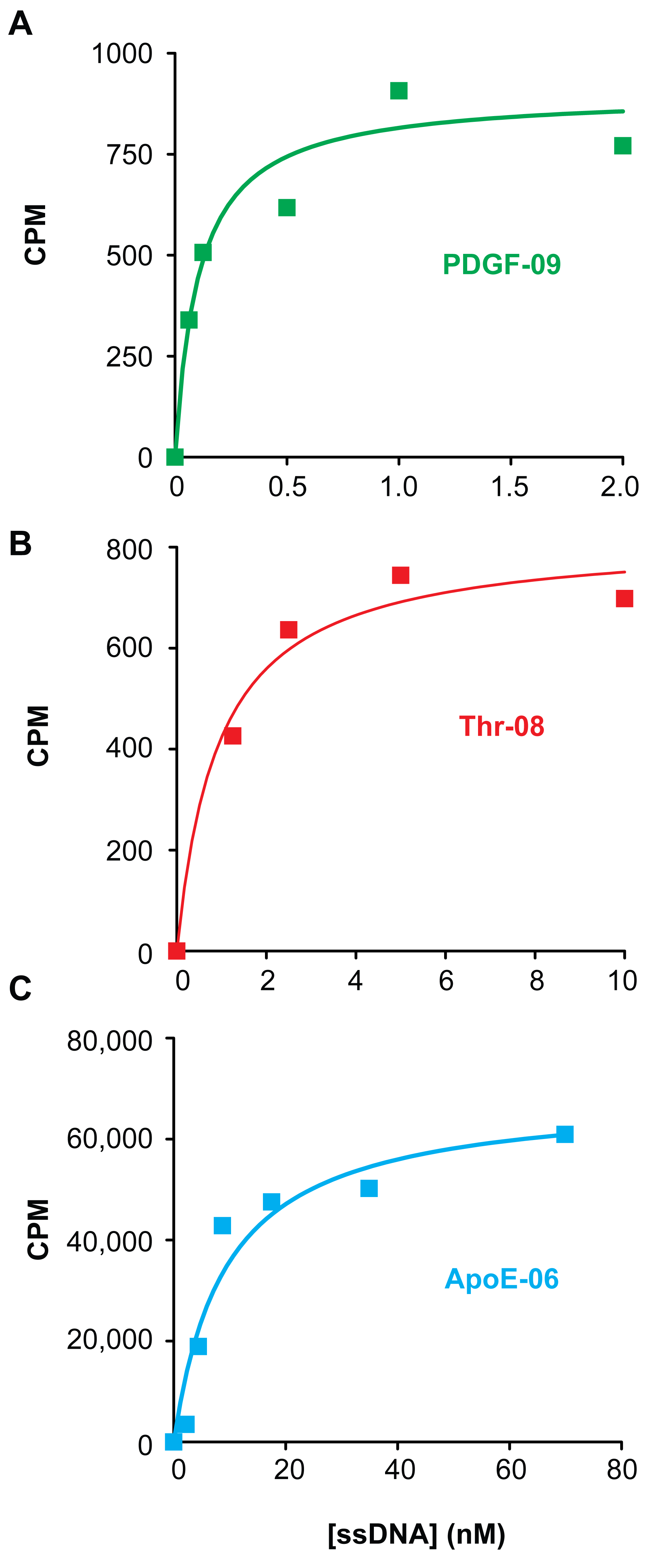

Supplement: Figure S3 — Affinity of selected radiolabeled aptamers based on filter binding. We determined the binding affinity of the three selected aptamers by a filter binding assay. We measured Kds for PDGF-BB-, thrombin- and ApoE-binding aptamers of 0.106 nM (A), 0.936 nM (B) and 9.25 nM (C), respectively. (TIF) [file pone.0027051.s003.tif]

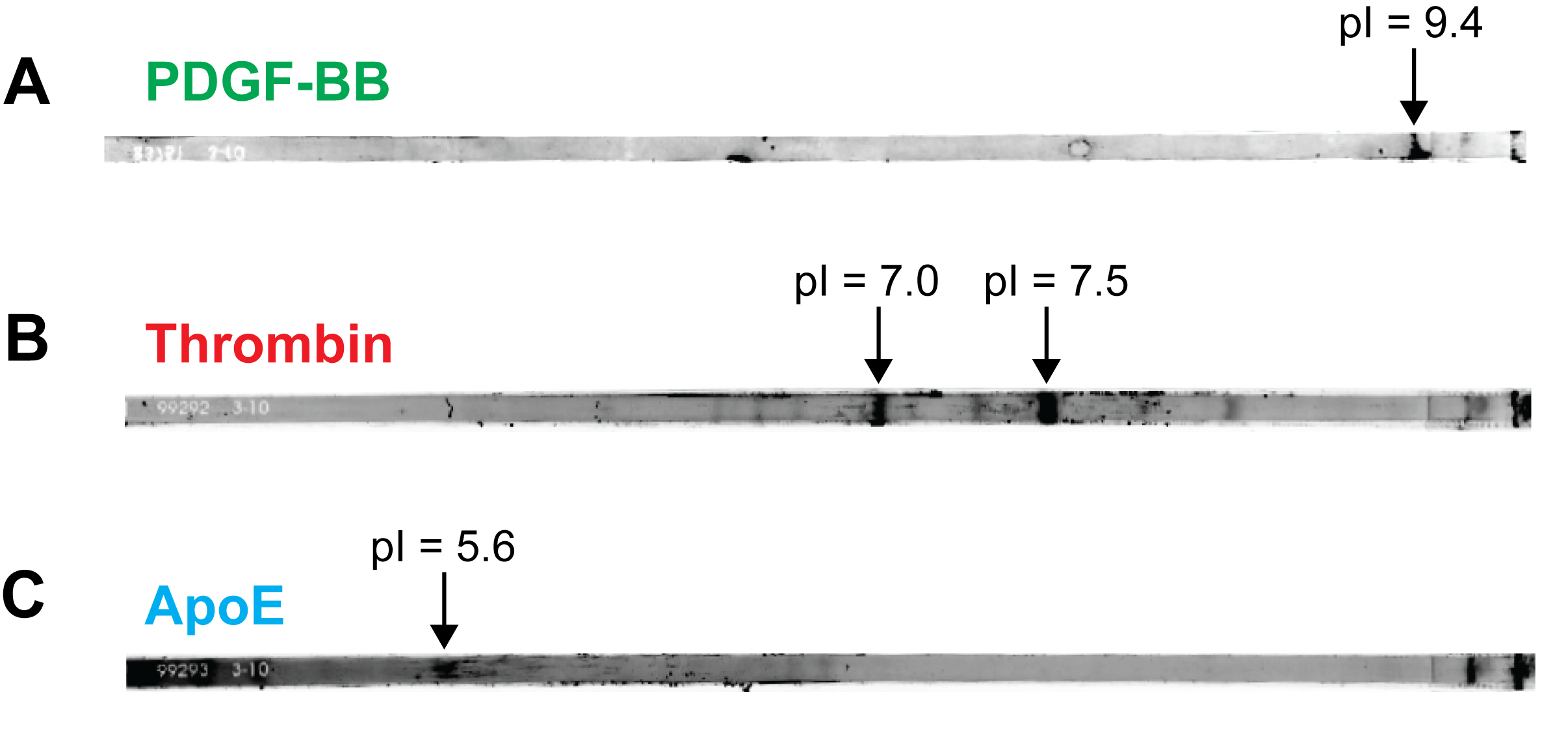

Supplement: Figure S4 — Measuring the isoelectric points (pIs) of PDGF-BB, thrombin and ApoE. We used isoelectric focusing (IEF) to measure target protein pI directly for (A) PDGF-BB, (B) thrombin and (C) ApoE under denaturing conditions for comparison against calculated pIs. pIs were determined based on band distance from the anodic end of the gel after focusing. For thrombin, we observed two main bands in the region from 7 to 7.5, in agreement with previous results [20]. (TIF) [file pone.0027051.s004.tif]

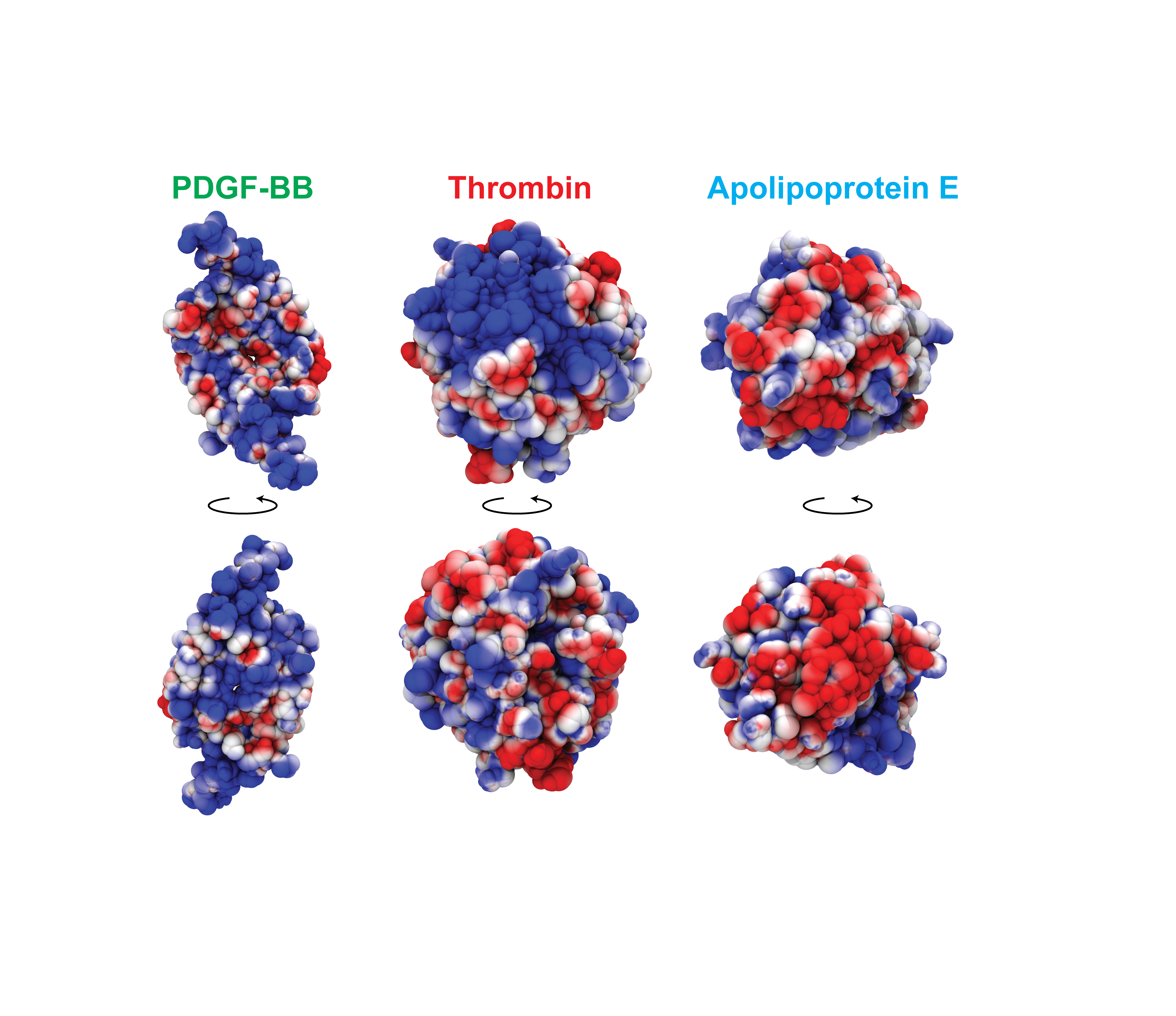

Supplement: Figure S5 — Electrostatic potential surfaces of PDGF-BB, thrombin and ApoE. The electrostatic potentials are mapped to the van der Waals surfaces of these three proteins. The bottom set of views show the proteins rotated 180° about the vertical axis. These three maps suggest a structural basis for the relationship between charge and binding affinity. Reflecting its high net positive charge (z = +15), a substantial portion of the PDGF-BB homodimer surface has a highly positive surface potential. These regions present sizable areas for possible interactions with the highly negatively charged DNA aptamers, and were identified as the actual binding sites for a previously-isolated PDGF-AB aptamer through cross-linking experiments [9]. Thrombin (z = +2.6) contains two somewhat smaller distinct regions of positive surface potential, and these were also identified as the binding sites of two previously-published thrombin aptamers [10], [11]. The surface potential of ApoE (z = −4.3) is more heterogeneous, reducing the area available for aptamer binding. These data support the view that aptamers often preferentially bind to positively charged surface patches, as has been observed in crystal structures of aptamers bound to proteins [21], [22], [23], [24], [25], [26]. (TIF) [file pone.0027051.s005.tif]

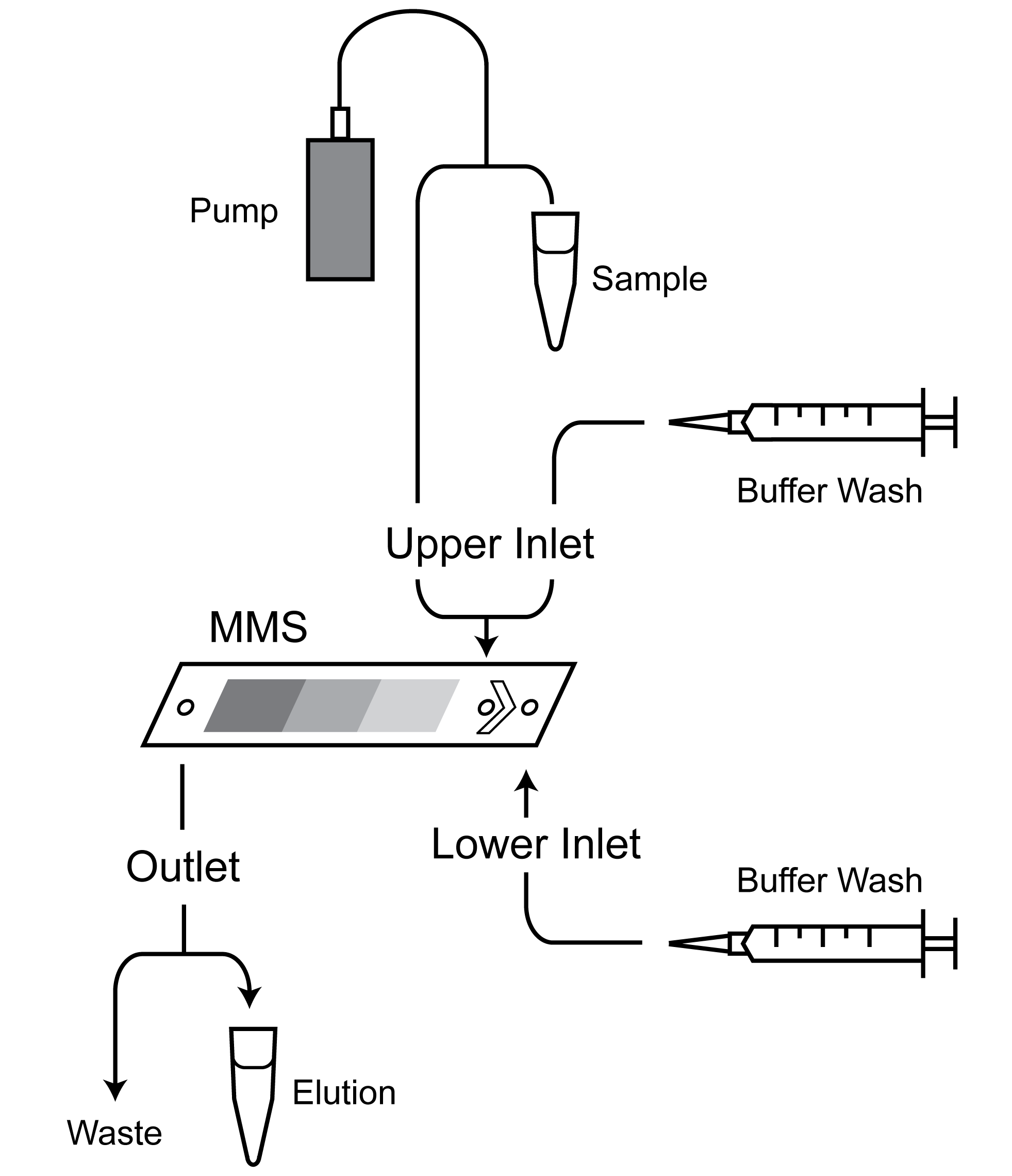

Supplement: Figure S6 — Schematic of micro-magnetic separation (MMS) device and pump configuration. The organization of the fluidic connections between the pumps and MMS chip are shown. Syringe pumps (not shown) were used to infuse buffer solution. (TIF) [file pone.0027051.s006.tif]
