# Supplementary material for: Probing the Limits of Aptamer Affinity with a Microfluidic SELEX Platform
Source: PLoS One. 2011 Nov 14;6(11):e27051. doi: 10.1371/journal.pone.0027051 (PMC3215713; doi:10.1371/journal.pone.0027051)
Supplement: Table S1 — Selected sequences. 60-mer random regions cloned from each selected round 3 pool. (DOCX) [file pone.0027051.s007.docx]

| **Clone** | **Selected region ( 5' to 3')** |
| --- | --- |
|  |  |
| PDGF-01 | CACGCGTACAAGTTGGGTGGAAGCATAGGCAATGAGCTCTCATTGGGTTACCTTTAAGGT |
| PDGF-02 | GTGACGTGAGCATCATGTTAATGAACCTTACCTAAAGGCACTGTCACACAGTTTAGACAT |
| PDGF-03 | TAGGAAATCTGGGTCATTGTTTCGAGATAGTATAATTTTGAATATAAGCTAAGAAATAGT |
| PDGF-04 | TTATGGGCCGCTTCATTTGACGCCGTTGTACTGGTTTAGGGCAACGGTTGAAATCCGCTC |
| PDGF-05 | AGGCGGTAAAGTACTCTTGCCAGAGGGAATTTTACTGGTCACTAAAAAATAGTCCGATTT |
| PDGF-06 | TGCTCGTCAAAGATAATGTACGCATCAGGCGCGATAATAAATCGTGAGCATCGTTTTGAT |
| PDGF-07 | GGAAGGCAGATTAAGTCGGTACTTTATTTGGTCATTAATGCTCATTAGTCGTTCTCCTGC |
| PDGF-08 | GTTGCATATTGAGCATCGTGTAATGATCCTGCAAAGGCATTATGCATCGGGTCTTCCT |
| PDGF-09 | GAATCATGATCCCCCATGACCTGGGGGCGTTACTGGCGAGCATCATGAGAGATCGTGCGA |
| PDGF-10 | ATGAGCATCGGGTTCCGTTAGAGTCTGATCCGGCAGGGTTCCTGGCGGCGTCGCTGAAT |
| PDGF-11 | TGTTTTGGTCACTGTCCCTTACTTTAAGTTTTAATGATAGTCCCTTTGGGCAATAGCTGC |
| PDGF-12 | AGCATCGGATATACAGAAACCCATATCGGATCCTGCATAGTTTATAGAGTAGGCAACTAA |
| PDGF-13 | GTCACTATATTTGATATTTAGCTTATAGTCCTGTAAAGGCATCGTCGTCATGATGACATC |
| PDGF-14 | TGGGGTAAGCGTTGATTGCATGGATAGGTAACCTTGCGTTATGTAATCCCTGTTGGG |
| PDGF-15 | TGTCTTAAGAGAGGATACGACATAAGATCATAGGGAGTAGGATCAAGTTGCTGAGGTCAT |
| PDGF-16 | GTTCCGTTTAGGGCATGGACGCACTATTAACGTTGCTAACATGGGACCCGTATGAGCTCG |
| PDGF-17 | AAGTCCCGTAGGGTATTTACTAAGAGCGAAATGAGTCCGTGAGTTAATTGTCATTTCATC |
| PDGF-18 | GGTCTTCCGCATATTGGAGACGCCGTGATGCTCTGTCAATAGAAGGCGGTACTTTAGGAA |
| PDGF-19 | TTGTACGTAGTCCCTCCGGGCATATAGAGTTGGTTATATGGTCATTGCGTTCATCCTTTG |
| PDGF-20 | GGTTTTAACTGATTTTCTCGGACCAAGGTTTCCGGGATTGTTAACTACGTACCAGTGTTC |
|  |  |
| Thr-01 | CAGCGCTAGGGCTTTTAGCGTAATGGGTAGGGTGGTGCGGTGCAGATATCGGAATTGGTG |
| Thr-02 | TTGAAGTAATTTTATAGGTCTTTTATTGGGTAGGGTGGTTTTAATTGGTGTGACAAATTG |
| Thr-03 | TTGAAGTAATTTTATAGGTTTTTTATTGGGTAGGGTGGTTTTAATTGGTGTGACAAATTG |
| Thr-04 | TTGAAGTAATTTTATAGGTTTTTTATTGGGTAGGGTGGTTTTAATTGGTGTGACAAATTG |
| Thr-05 | CAGCGCTAGGGCTTTTAGCGTAATGGGTAGGGTGGTGCGGTGCAGATATCGGAATTGGTG |
| Thr-06 | CAGCGCTAGGGCTTTTAGCGTAATGGGTAGGGTGGTGCGATGCAGATATCGGAATTGGTG |
| Thr-07 | CAGCGCTAGGGCTTTTAGCGTAATGGGTAGGGTGGTGCGGTGCAGATATCGGAATTGGTG |
| Thr-08 | CAGCGCTAGGGCTTTTAGCGTAATGGGTAGGGTGGTGCGGTGCAGATATCGGAATTGGTG |
| Thr-09 | CAGCGCTAGGGCTTTTAGCGTAATGGGTAGGGTGGTGCGGTGCAGATATCGGAATTGGTG |
| Thr-10 | CAGCGCTAGGGCTTTTAGCGTAATGGGTAGGGTGGTGCGGTGCAGATATCGGAATTGGTG |
| Thr-11 | CAGCGCTAGGGCTTTTAGCGTAATGGGTAGGGTGGTGCGGTGCAGATATCGGAATTGGTG |
| Thr-12 | TTGAAGTAATTTTATAGGTTTTTTATTGGGTAGGGTGGTTTTAATTGGTGTGACAAATTG |
| Thr-13 | CCGGGGCATGACAACCTATTCGGCTCTCGGCTCATGTCGTATGGTTTAGTGTGGGAGGTG |
| Thr-14 | TTGAAGTAATTTTATAGGTTTTTTATTGGGTAGGGTGGTTTTAATTGGTGTGACAAATTG |
| Thr-15 | TTGAAGTAATTTTATAGGTTTTTTATTGGGTAGGGTGGTTTTAATTGGTGTGACAAATTG |
| Thr-16 | TTATGGGTAGGGTGGTGTGTTTGGCGCGAGCCATTTTCAGTAGGTAATCTCGACCTTTTG |
| Thr-17 | TTGAAGTAATTTTATAGGTTTTTTATTGGGTAGGGTGGTTTTAATTGGTGTGACAAATTG |
| Thr-18 | GTTGGGTTGCGTAGACGACGTTAGACTAGAGTGATAGTTTGGAGCCAGGAAACTGGACTT |
| Thr-19 | CAGCGCTAGGGCTTTTAGCGTAATGGGTAGGGTGGTGCGGTGCAGATATCGGAATTGGTG |
| Thr-20 | TTGAAGTAATTTTATAGGTTTTTTATTGGGTAGGGTGGTTTTAATTGGTGTGACAAATTG |
|  |  |
| ApoE-01 | GGAAAACGATGTAGCTATATCAAATCCTCCGCTGTTGGGCTAATTACGCTCCATCCCACC |
| ApoE-02 | TATAGTGAAATGAGAGTCGGCAAGCGTTCCGATCCCTTTTGACTGTTTCCAACTGCTCGC |
| ApoE-03 | CGTCTAAATTCGTTATTTATAAAGCATTCATTTGCATGCGGGTCGAGTGTAGGAAATTCC |
| ApoE-04 | CGGTGTGTGGTACGGCGTTCGTTGGGTGGTGGTAAATGCGATCGGTACGGATGTGGTCTC |
| ApoE-05 | CTAGGCTCTCAGAGAGGTTTGAAACAAGGAACCATAATCCGGCTGTTCAAGAGTAATTTT |
| ApoE-06 | ACTAGCTACGGGGTGGGTGGGCGGTGTCAGTTTGTTTATTGGTGCTATACATCCTCTATA |
| ApoE-07 | ACCATTTTGGAAAGAATTGAAGTTTAGTTGGGTCCAGGGAACTTTTGACCCCGCCGAATC |
| ApoE-08 | CGGTACTACAGGTCCATAGCCTCAATCTCCCGCACTAATTTCCTAGGGAGGAAAGTGCAC |
| ApoE-09 | AGAGTGTAACTATTAGACAAACACCTTTTTAGTACTTGTGGCTTTCTTTTTTGTTTGAAT |
| ApoE-10 | TGGAAGTGTCATTTGGTCGTGTGGGATGCCTTTTAGGGAAGAGGTGTCAGTGTGCGGGAT |
| ApoE-11 | ATCCTCGTTTCTATTCAGGGTGCACAGTCTCTGGTGATTTTTGGGTACCTTGTTACCTCT |
| ApoE-12 | CTTCACAAACGCACAGTACAGGGTAAAGTAGATTTTGTTTTGATTTAGGTCACGGGCGGT |
| ApoE-13 | AGCGCCATAGGGAGTCACATAGGGGAAGGGGAGGAAGATTGGTAGCGTGGATCCGAGGGT |
| ApoE-14 | AAAGCATCGTGGCCCTTCTAGGAAAAAAAAACAACGCATAATCTCTTAGCGCGGTCCGGT |
| ApoE-15 | CAGCACCCGAAATGTGAATAGTTCGTTTTCGCTATACCCATGCATACCCAGTGCGAGGCA |
| ApoE-16 | GAAACGATGCTCTGGCTCTACGGGTATTAGCTTTCCCTTAGAAAGGATATGCGAACGGCA |
| ApoE-17 | CTGCGCGTCTTGGTGGTCGAATGCTGGAGCACTGTCGTGCGATTGCCGGTTGCCTTTGTA |
| ApoE-18 | CGCTTCCTCGTTCGTTCCGGTGGTCTAAGGTTTTTAACTTACTGGGGTTGCTAACTAGAT |
| ApoE-19 | CTCCCTTCCGAGAACGTTGTAACAAGTAAGCATACATTGGTAGACGTATTGATTAGGGGG |
| ApoE-20 | TTTGGGGGCGACCTCCACTGTCTGCGGTGTGGCGTACGCGGAGAGCGGCGAGGACCTCTT |
