# Supplementary material for: Probing the Limits of Aptamer Affinity with a Microfluidic SELEX Platform
Source: PLoS One. 2011 Nov 14;6(11):e27051. doi: 10.1371/journal.pone.0027051 (PMC3215713; doi:10.1371/journal.pone.0027051)
Supplement: Table S2 — Sequences of previously published aptamers. We synthesized these aptamer sequences with a 5′-FAM fluorophore and assayed their binding affinity using the bead-based method. (DOCX) [file pone.0027051.s008.docx]

| **Citation** | **Identification** | **Sequence** |
| --- | --- | --- |
|  |  |  |
| Green et al., 1996 [9] | 36t | CACAGGCTACGGCACGTAGAGCATCACCATGATCCTGTG |
| Bock et al., 1992 [10] | Consensus 15-mer | GGTTGGTGTGGTTGG |
| Tasset et al., 1997 [11] | 60-18[29] | AGTCCGTGGTAGGGCAGGTTGGGGTGACT |
|  |  |  |
