# Supplementary material for: Probing the Limits of Aptamer Affinity with a Microfluidic SELEX Platform
Source: PLoS One. 2011 Nov 14;6(11):e27051. doi: 10.1371/journal.pone.0027051 (PMC3215713; doi:10.1371/journal.pone.0027051)
Supplement: Table S3 — A list of 75 previously published aptamers. This list was compiled from PubMed searches and includes both RNA and DNA aptamers against protein targets. Where not provided in the reference, isoelectric points were determined by UniProt (www.uniprot.org) and ExPASy (www.expasy.org). (DOCX) [file pone.0027051.s009.docx]

| **Target** | **pI** | **Kd** | **Lead Author** | **Year** |
| --- | --- | --- | --- | --- |
|  |  |  |  |  |
| 14-3-3γ | 4.80 | 560 | Stevenson | 2008 |
| Angiogenin | 9.70 | 5 | Bock | 2004 |
| Angiopoietin-2 | 5.41 | 3 | White | 2003 |
| Anthrax protective antigen | 5.64 | 112 | Cella | 2010 |
| Basic fibroblast growth factor | 9.60 | 0.08 | Bock | 2004 |
| B-secretase cytoplasmic domain | 6.74 | 280 | Rentmeister | 2006 |
| C-reactive protein | 5.30 | 150 | Bock | 2004 |
| C-reactive protein | 5.45 | 3.51 | Huang | 2010 |
| Calf intestinal alkaline phosphatase | 6.29 | 0.26 | Hicke | 1999 |
| CD4 antigen | 9.60 | 0.5 | Kraus | 1998 |
| CED-9 (bcl-2) | 8.57 | 4 | Yang | 2006 |
| Complement factor C5 | 6.11 | 30 | Biesecker | 1999 |
| CTLA-4 (mouse) | 4.85 | 10 | Santulli-Marotto | 2003 |
| Drosophila B52 | 11.40 | 20 | Shi | 1999 |
| Endostatin | 9.30 | 0.4 | Bock | 2004 |
| Eotaxin | 9.90 | 0.5 | Bock | 2004 |
| ERK2 | 6.53 | 1.3 | Seiwert | 2000 |
| HCV 3a polymerase | 9.25 | 1.3 | Jones | 2006 |
| HCV NS3 | 9.33 | 6.3 | Fukuda | 2003 |
| HCV NS5B | 9.25 | 1.5 | Biroccio | 2002 |
| HER3 | 6.11 | 45 | Chen | 2003 |
| HGF | 8.20 | 1 | Bock | 2004 |
| HIV Gag p55 | 9.83 | 1 | Lochrie | 1997 |
| HIV Gag p55 | 9.83 | 80 | Ramalingam | 2010 |
| HIV gp120 | 9.22 | 171 | Sayer | 2002 |
| HIV gp120 | 9.22 | 52 | Zhou | 2009 |
| HIV-1 integrase | 8.16 | 12 | Allen | 1995 |
| Human nonpancreatic secretory phospholipase A2 | 10.40 | 1.7 | Bridonneau | 1998 |
| Hut operon positive regulatory protein Bacillus subtilis | 5.97 | 57 | Kumarevel | 2004 |
| IFN gamma | 9.82 | 1.8 | Kubik | 1997 |
| IgE | 5.50 | 6 | Wiegand | 1996 |
| IL-12 | 5.70 | 2 | Bock | 2004 |
| IL-16 | 4.80 | 0.01 | Bock | 2004 |
| IL-17A | 8.62 | 0.0485 | Ishiguro | 2010 |
| IL-6 sRa | 8.60 | 6 | Bock | 2004 |
| IL-8 | 9.20 | 300 | Bock | 2004 |
| IP-10 | 10.20 | 4 | Bock | 2004 |
| IP-10 | 9.97 | 1.6 | Marro | 2005 |
| I-TAC | 10.00 | 1 | Bock | 2004 |
| Keratinocyte growth factor (KGF) | 9.25 | 0.0003 | Pagratis | 1997 |
| L-Selectin | 6.04 | 1.8 | Hicke | 1996 |
| Lysozyme | 9.36 | 31 | Cox | 2001 |
| Lysozyme | 9.32 | 2.8 | Tran | 2010 |
| Monocyte chemoattractant protein-1 (MCP-1) | 9.39 | 0.18 | Rhodes | 2001 |
| MutS (T thermophilus) | 6.63 | 15 | Drabovich | 2005 |
|  |  |  |  |  |

Continued-

| **Target** | **pI** | **Kd** | **Lead Author** | **Year** |
| --- | --- | --- | --- | --- |
|  |  |  |  |  |
| NF-κB | 5.46 | 11 | Wurster | 2008 |
| Oncostatin M | 9.97 | 7 | Rhodes | 2000 |
| Osteopontin | 4.35 | 18 | Mi | 2008 |
| Papillomavirus 16 E7 | 4.20 | 87 | Nicol | 2011 |
| PDGF-BB | 9.38 | 0.1 | Green | 1996 |
| Pepocin | 9.90 | 17.9 | Hirao | 2000 |
| Plasminogen activating factor I | 6.68 | 0.177 | Blake | 2009 |
| Plasminogen activating factor I | 6.68 | 1.23 | Madsen | 2010 |
| Protein Kinase C | 6.57 | 7 | Conrad | 1994 |
| Protein Kinase Delta | 7.93 | 122 | Mallikaratchy | 2006 |
| P-selectin | 4.11 | 0.016 | Jenison | 1998 |
| RAF-1 | 9.33 | 152 | Kimoto | 2002 |
| rHuEPO-α | 8.75 | 82 | zhang | 2010 |
| Ricin A-chain | 6.14 | 7.4 | Hesselberth | 2000 |
| RUNX1 (AML1) | 9.40 | 95 | Barton | 2009 |
| Sclerostin | 9.57 | 200 | Shum | 2011 |
| Secretory phospholipase A2 (sPLA2) | 9.40 | 0.12 | Bridonneau | 1998 |
| Streptavidin | 6.00 | 70 | Srisawat | 2001 |
| Streptavidin | 6.00 | 7 | Tahiri-Alaoui | 2002 |
| Streptavidin | 6.00 | 85 | Stoltenburg | 2005 |
| TATA binding protein | 9.40 | 2 | Shi | 2007 |
| Thrombin | 8.32 | 4 | Tasset | 1995 |
| Thrombin (bovine) | 8.32 | 164 | Liu | 1999 |
| TIMP-1 | 8.50 | 0.1 | Bock | 2004 |
| Toll like receptor-2 | 6.14 | 0.028 | Chang | 2009 |
| Transforming growth factor-beta 2 receptor (TGF-β2R) | 5.60 | 1.52 | Ohuchi | 2006 |
| VEGF | 9.49 | 0.14 | Green | 1995 |
| VEGF | 9.49 | 0.002 | Ruckman | 1998 |
| VEGF | 9.20 | 0.3 | Bock | 2004 |
| Von willebrand factor | 5.75 | 2 | Diener | 2009 |
|  |  |  |  |  |
